# Induction of COX-2-PGE2 synthesis by activation of the MAPK/ERK pathway contributes to neuronal death triggered by TDP-43-depleted microglia

**DOI:** 10.1038/cddis.2015.69

**Published:** 2015-03-26

**Authors:** Q Xia, Q Hu, H Wang, H Yang, F Gao, H Ren, D Chen, C Fu, L Zheng, X Zhen, Z Ying, G Wang

**Affiliations:** 1 Laboratory of Molecular Neuropathology, Jiangsu Key Laboratory of Translational Research and Therapy for Neuro-Psycho-Diseases and College of Pharmaceutical Sciences, Soochow University, Suzhou, Jiangsu, China; 2 Key Laboratory of Brain Function and Disease, School of Life Sciences, University of Science & Technology of China, Chinese Academy of Sciences, Hefei, Anhui, China; 3 Jiangsu Key Laboratory of Preventive and Translational Medicine for Geriatric Diseases, College of Pharmaceutical Sciences, Soochow University, Suzhou, Jiangsu, China

## Abstract

Neuroinflammation is a striking hallmark of amyotrophic lateral sclerosis (ALS) and other neurodegenerative disorders. Previous studies have shown the contribution of glial cells such as astrocytes in TDP-43-linked ALS. However, the role of microglia in TDP-43-mediated motor neuron degeneration remains poorly understood. In this study, we show that depletion of TDP-43 in microglia, but not in astrocytes, strikingly upregulates cyclooxygenase-2 (COX-2) expression and prostaglandin E2 (PGE2) production through the activation of MAPK/ERK signaling and initiates neurotoxicity. Moreover, we find that administration of celecoxib, a specific COX-2 inhibitor, greatly diminishes the neurotoxicity triggered by TDP-43-depleted microglia. Taken together, our results reveal a previously unrecognized non-cell-autonomous mechanism in TDP-43-mediated neurodegeneration, identifying COX-2-PGE2 as the molecular events of microglia- but not astrocyte-initiated neurotoxicity and identifying celecoxib as a novel potential therapy for TDP-43-linked ALS and possibly other types of ALS.

Amyotrophic lateral sclerosis (ALS) is an adult-onset neurodegenerative disease characterized by the degeneration of motor neurons in the brain and spinal cord.^1^ Most cases of ALS are sporadic, but 10% are familial. Familial ALS cases are associated with mutations in genes such as Cu/Zn superoxide dismutase 1 (*SOD1*), TAR DNA-binding protein 43 (*TARDBP*) and, most recently discovered, *C9orf72.* Currently, most available information obtained from ALS research is based on the study of *SOD1*, but new studies focusing on *TARDBP* and *C9orf72* have come to the forefront of ALS research.^1, 2^ The discovery of the central role of the protein TDP-43, encoded by *TARDBP*, in ALS was a breakthrough in ALS research.^3, 4, 5^ Although pathogenic mutations of TDP-43 are genetically rare, abnormal TDP-43 function is thought to be associated with the majority of ALS cases.^1^ TDP-43 was identified as a key component of the ubiquitin-positive inclusions in most ALS patients and also in other neurodegenerative diseases such as frontotemporal lobar degeneration,^6, 7^ Alzheimer's disease (AD)^8, 9^ and Parkinson's disease (PD).^10, 11^ TDP-43 is a multifunctional RNA binding protein, and loss-of-function of TDP-43 has been increasingly recognized as a key contributor in TDP-43-mediated pathogenesis.^5, 12, 13, 14^

Neuroinflammation, a striking and common hallmark involved in many neurodegenerative diseases, including ALS, is characterized by extensive activation of glial cells including microglia, astrocytes and oligodendrocytes.^15, 16^ Although numerous studies have focused on the intrinsic properties of motor neurons in ALS, a large amount of evidence showed that glial cells, such as astrocytes and microglia, could have critical roles in SOD1-mediated motor neuron degeneration and ALS progression,^17, 18, 19, 20, 21, 22^ indicating the importance of non-cell-autonomous toxicity in SOD1-mediated ALS pathogenesis.

Very interestingly, a vital insight of neuroinflammation research in ALS was generated by the evidence that both the mRNA and protein levels of the pro-inflammatory enzyme cyclooxygenase-2 (COX-2) are upregulated in both transgenic mouse models and in human postmortem brain and spinal cord.^23, 24, 25, 26, 27, 28, 29^ The role of COX-2 neurotoxicity in ALS and other neurodegenerative disorders has been well explored.^30, 31, 32^ One of the key downstream products of COX-2, prostaglandin E2 (PGE2), can directly mediate COX-2 neurotoxicity both *in vitro* and *in vivo*.^33, 34, 35, 36, 37^ The levels of COX-2 expression and PGE2 production are controlled by multiple cell signaling pathways, including the mitogen-activated protein kinase (MAPK)/ERK pathway,^38, 39, 40^ and they have been found to be increased in neurodegenerative diseases including AD, PD and ALS.^25, 28, 32, 41, 42, 43, 44, 45, 46^ Importantly, COX-2 inhibitors such as celecoxib exhibited significant neuroprotective effects and prolonged survival or delayed disease onset in a SOD1-ALS transgenic mouse model through the downregulation of PGE2 release.^28^

Most recent studies have tried to elucidate the role of glial cells in neurotoxicity using TDP-43-ALS models, which are considered to be helpful for better understanding the disease mechanisms.^47, 48, 49, 50, 51^ Although the contribution of glial cells to TDP-43-mediated motor neuron degeneration is now well supported, this model does not fully suggest an astrocyte-based non-cell autonomous mechanism. For example, recent studies have shown that TDP-43-mutant astrocytes do not affect the survival of motor neurons,^50, 51^ indicating a previously unrecognized non-cell autonomous TDP-43 proteinopathy that associates with cell types other than astrocytes.

Given that the role of glial cell types other than astrocytes in TDP-43-mediated neuroinflammation is still not fully understood, we aim to compare the contribution of microglia and astrocytes to neurotoxicity in a TDP-43 loss-of-function model. Here, we show that TDP-43 has a dominant role in promoting COX-2-PGE2 production through the MAPK/ERK pathway in primary cultured microglia, but not in primary cultured astrocytes. Our study suggests that overproduction of PGE2 in microglia is a novel molecular mechanism underlying neurotoxicity in TDP-43-linked ALS. Moreover, our data identify celecoxib as a new potential effective treatment of TDP-43-linked ALS and possibly other types of ALS.

## Results

### Selective upregulation of COX-2 in TDP-43-deficient microglia but not astrocytes

**Figure 1:**
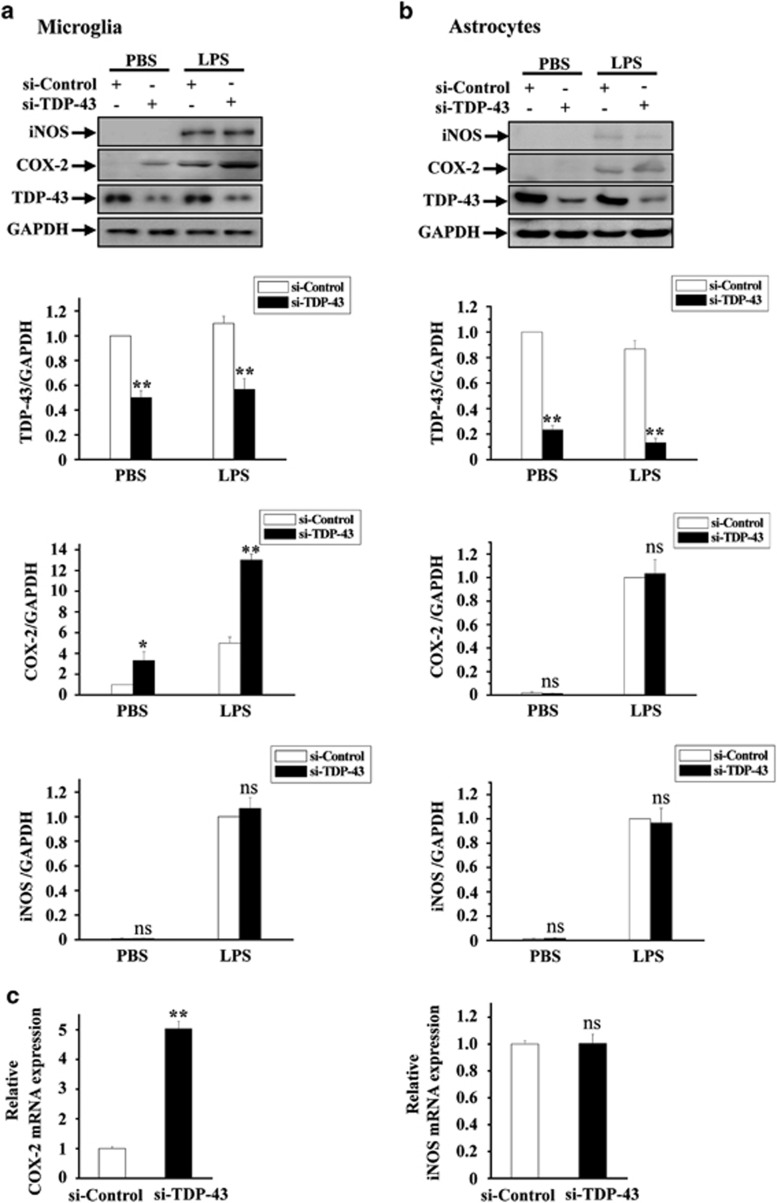
TDP-43 specifically regulates the expression level of COX-2, but not iNOS, in microglia. (**a**) Primary cultured microglia were transfected with si-control and si-TDP-43. After 72 h, the cells were treated with PBS or LPS (0.1 μg/ml) for 24 h, and then the cell lysates were subjected to immunoblot analysis using antibodies targeting iNOS, COX-2, TDP-43 and GAPDH. The quantification of TDP-43, COX-2 and iNOS levels is shown in the lower panels, representing three independent experiments. The data are presented as the means±S.E.M.; ns, not significantly different; **P<0.01; one-way ANOVA. (**b**) Experiments similar to those in (**a**) were performed in primary cultured astrocytes. The quantification of TDP-43, COX-2 and iNOS levels is shown on the lower side. The data from three independent experiments are presented as the means±S.E.M.; ns, not significantly different; **P<0.01; one-way ANOVA. (**c**) Primary cultured microglia were transfected with si-control and si-TDP-43 and cultured for 96 h, and then the cells were processed for qRT-PCR. The mRNA levels of COX-2 and iNOS were each quantified and normalized to GAPDH. The data from three independent experiments are shown as the means±S.E.M., ns, not significantly different; **P<0.01; one-way ANOVA

The goal of this study is to better understand the role of TDP-43 in neuroinflammation, a key feature of ALS. To gain insight into the roles of microglia and astrocytes in TDP-43-mediated neurotoxicity, we knocked down TDP-43 in primary cultured microglia and astrocytes using siRNA against TDP-43, and we then used biochemical approaches to detect the expression levels of COX-2 and iNOS, two markers of neuroinflammation. RNAi-mediated depletion of TDP-43 in microglia resulted in a greatly increased protein level of COX-2 with or without LPS treatment, whereas iNOS protein level was unchanged (Figure 1a). In contrast, protein levels of either COX-2 or iNOS were not increased in TDP-43-depleted astrocytes with or without LPS treatment (Figure 1b). To further explore the effects of regulating TDP-43 on the expression of COX-2 and iNOS, we performed quantitative real-time PCR (qRT-PCR) assays: the mRNA level of COX-2, but not iNOS, was remarkably upregulated in TDP-43-depleted microglia (Figure 1c). Similar results were obtained in BV2 cells (microglial cell line) as in primary cultured microglia (Supplementary Figure S1). Taken together, these results indicate a critical role for TDP-43 in COX-2 expression in microglia.

### TDP-43 specifically regulates COX-2 expression in a MAPK/ERK-dependent manner

**Figure 2:**
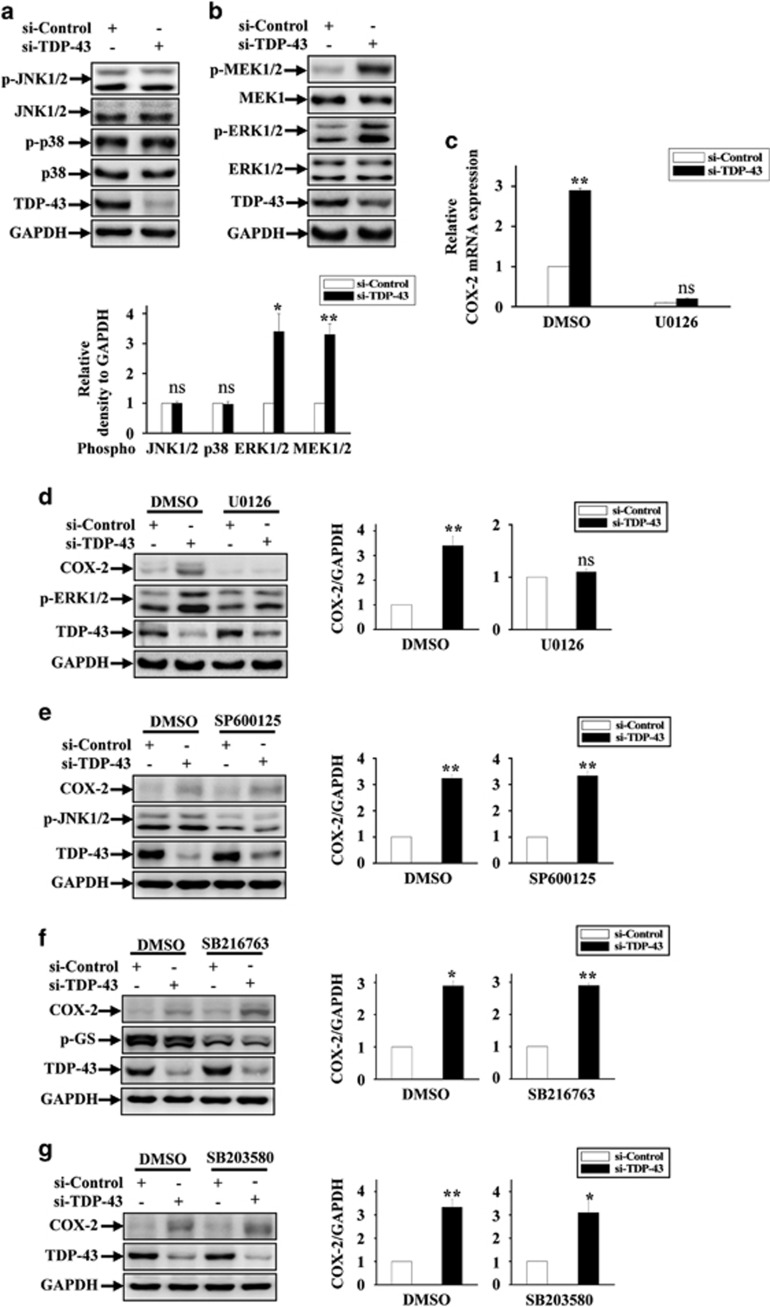
Loss of TDP-43 enhanced COX-2 expression by specifically activating MAPK/ERK signaling. (**a**) BV2 cells were transfected with si-control and si-TDP-43. At 72 h after transfection, the cell lysates were subjected to immunoblot analysis using anti-p-JNK1/2, JNK1/2, p-p38, p38, TDP-43 and GAPDH antibodies. The quantification data are shown on the lower side. The data from three independent experiments are presented as the means±S.E.M.; ns, not significantly different; one-way ANOVA. (**b**) Similar experiments as in (**a**) were performed, but using anti-p-MEK1/2, MEK1, p-ERK1/2, ERK1/2, TDP-43 and GAPDH antibodies. The relative densities are shown on the lower side. The data from three independent experiments are indicated as the means±S.E.M.; ns, not significantly different; *P<0.05; **P<0.01; one-way ANOVA. (**c**) BV2 cells were transfected with si-control and si-TDP-43 and cultured for 48 h. Then, the cells were treated with DMSO or U0126 (20 *μ*M) for 24 h. Subsequently, the cells were processed for qRT-PCR analysis. The mRNA level of COX-2 was quantified and normalized to GAPDH. Data from three independent experiments are presented as the means±S.E.M.; ns, not significantly different; **P<0.01; one-way ANOVA. (**d**) Similar transfections and treatments as in (**c**) were performed in BV2 cells, and the cell lysates were subjected to immunoblot analysis using anti-COX-2, p-ERK1/2, TDP-43 and GAPDH antibodies. The relative densities are shown on the right side. The data from three independent experiments are presented as the means±S.E.M.; ns, not significantly different; **P<0.01; one-way ANOVA. (**e**–**g**) Similar experiments as in (**d**) were performed, but by incubating cells with SP600125 (10 *μ*M), SB216763 (10 *μ*M) and SB203580 (10 *μ*M) instead of U0126. The cell lysates were subjected to immunoblot analysis using anti-COX-2, TDP-43, GAPDH, p-JNK1/2 and p-GS antibodies. The relative densities are shown on the right side. The data from three independent experiments are presented as the means±S.E.M.; *P<0.05; **P<0.01; one-way ANOVA

To characterize the molecular mechanism underlying the upregulation of COX-2 in TDP-43-deficient microglia, we examined the changes in cell signaling pathways accompanied by upregulation of COX-2 expression in TDP-43-deficient microglia. Given that the classical MAPK signaling pathway could regulate COX-2 expression and is significantly associated with various neurodegenerative diseases, we hypothesized that TDP-43 might be involved in the regulation of MAPKs. To test this possibility, we detected the total and phosphorylated levels of MEK, ERK, JNK and p38 in TDP-43-deficient microglia. As shown in Figures 2a and b, depletion of TDP-43 markedly increased phosphorylated MEK and ERK but not phosphorylated JNK or p38. Meanwhile, total protein levels of JNK, p38, MEK and ERK were not changed.

Given that TDP-43 strongly enhanced MAPK/ERK activation and COX-2 expression, we hypothesized that TDP-43 might regulate COX-2 by targeting ERK. Relatedly, we found that both mRNA and protein levels of COX-2 were no longer increased in TDP-43-deficient microglia under treatment with U0126, a MEK1 inhibitor (Figures 2c and d). In contrast, treatments with a set of other inhibitors, SP600125 (JNK inhibitor), SB216763 (GSK3*β* inhibitor) and SB203580 (p38 inhibitor), had no effect on TDP-43-mediated COX-2 expression (Figures 2e and g). Therefore, our data demonstrate that TDP-43 regulates microglial COX-2 expression by specifically targeting MAPK/ERK and not other cell signaling pathways.

### TDP-43 regulates AP-1 transcriptional activity by targeting the MAPK/ERK pathway

**Figure 3:**
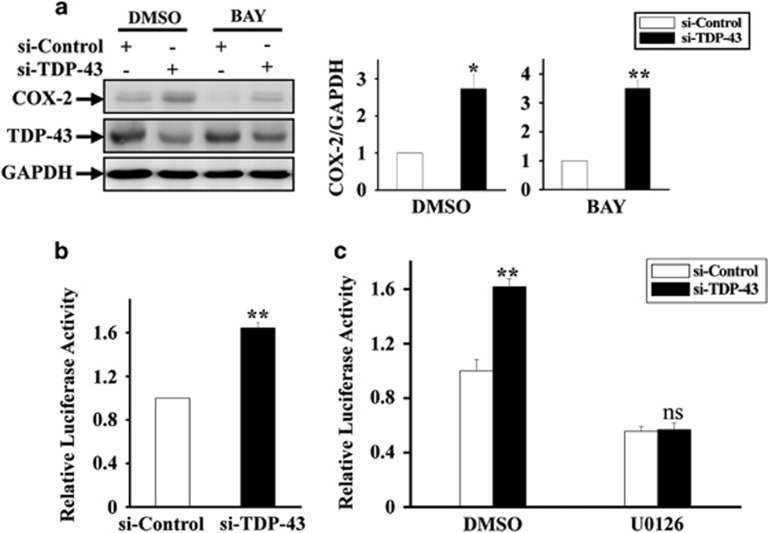
TDP-43 influences AP-1 transcriptional activity in a MAPK-signaling-dependent manner. (**a**) Experiments similar to those in Figure 2d were performed in BV2 cells, but by incubating cells with BAY (25 *μ*M) instead of U0126. The cell lysates were subjected to immunoblot analysis using anti-COX-2, TDP-43 and GAPDH antibodies. The relative densities are shown on the right side. The data from three independent experiments are presented as the means±S.E.M.; *P<0.05; **P<0.01; one-way ANOVA. (**b**) The stable BV2 AP-1 cell lines were transfected with si-control and si-TDP-43 for 72 h. Then, the expression of the AP-1 reporter gene was assessed. Data from three independent experiments are presented as the means±S.E.M.; **P<0.01; one-way ANOVA. (**c**) Similar experiments as in (**b**) were performed, and the cells were treated with DMSO or U0126 (20 *μ*M) for the last 24 h of the transfection. The data from three independent experiments are presented as the means±S.E.M.; ns, not significantly different; **P<0.01; one-way ANOVA

Among the various signaling molecules associated with COX-2 expression, NF-*κ*B and AP-1 are two transcription factors that have been known to act downstream of MAPK/ERK, and they can tightly regulate COX-2 expression. To identify which signaling pathway is involved in TDP-43-mediated COX-2 expression, we first employed an NF-*κ*B inhibitor, BAY. The protein expression level of COX-2 was still increased in TDP-43-deficient microglia treated with BAY (Figure 3a), suggesting that TDP-43 regulates COX-2 in an NF-*κ*B-independent manner. Next, we detected the transcriptional activity of AP-1 in TDP-43-depleted cells using a luciferase reporter gene assay. We found that depletion of TDP-43 significantly increased AP-1 transcriptional activity in microglia under normal conditions but not under treatment of U0126 (Figures 3b and c), suggesting that TDP-43 regulates AP-1 transcriptional activity in an ERK-dependent manner.

### TDP-43 controls the release of PGE2 in a MAPK/ERK- and COX-2-dependent manner

**Figure 4:**
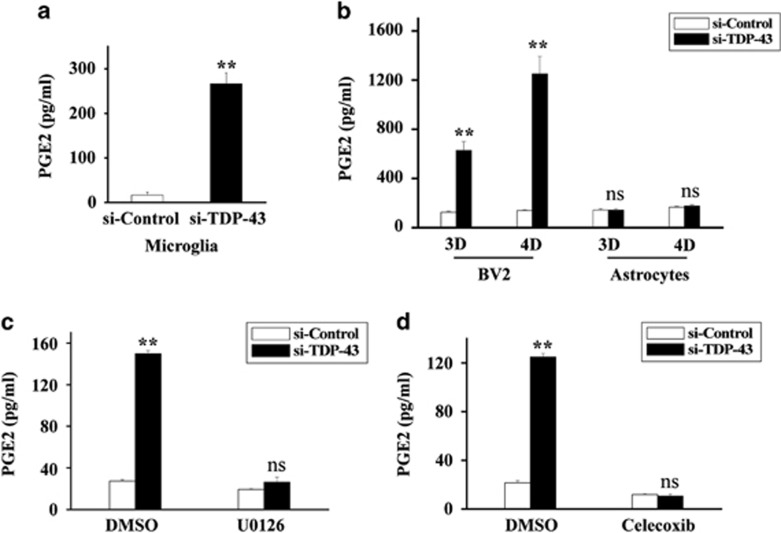
Loss of TDP-43 strikingly promotes the production of PGE2 in microglia but not in astrocytes. (**a**) Primary cultured microglia were transfected with si-control and si-TDP-43 for 96 h. Then, the media from for 24 h cultures of microglia were subjected to PGE2 enzyme-linked immunosorbent assays (ELISAs). The data from three independent experiments are presented as the means±S.E.M.; **P<0.01; one-way ANOVA (**b**) BV2 cells and primary cultured astrocytes were transfected with si-control or si-TDP-43 for 72 h or 96 h at a final density of 1 × 10^6^ cells per well in 12-well plates. Then, the 1 : 10 diluted media from 24 h cultures of BV2 cells or primary cultured astrocytes were subjected to PGE2 ELISAs. The data from three independent experiments are presented as the means±S.E.M.; ns, not significantly different; **P<0.01; one-way ANOVA. (**c**) BV2 cells were transfected with si-control or si-TDP-43 for 48 h. Then, the cells were treated with DMSO or U0126 (20 *μ*M) for 24 h, with a final cell density of 3 × 10^5^ cells per well in 24-well plates. Next, the media from 24 h cultures of BV2 cells were subjected to PGE2 ELISAs. The data from three independent experiments are presented as the means±S.E.M.; ns, not significantly different; **P<0.01; one-way ANOVA. (**d**) Similar experiments as in (**b**) were performed, but the BV2 cells were treated with celecoxib (50 *μ*M) instead of U0126. The data from three independent experiments are indicated as the means±S.E.M.; ns, not significantly different; **P<0.01; one-way ANOVA

Because COX-2 activity is strongly associated with the production of PGE2, a key downstream product of COX-2, we next investigated whether TDP-43 could regulate the production of PGE2. We found that depletion of TDP-43 greatly increased the release of PGE2 from microglia, but not from astrocytes, in a time-dependent manner (Figures 4a and b, and Supplementary Figure S2). However, this effect of TDP-43 was blocked by incubating the microglia with either U0126 or celecoxib, a specific COX-2 inhibitor (Figures 4c and d). Taken together, our results suggest a key role for COX-2 in TDP-43-mediated regulation of PGE2.

### Loss of TDP-43 induces selective microglia-mediated neuronal death

**Figure 5:**
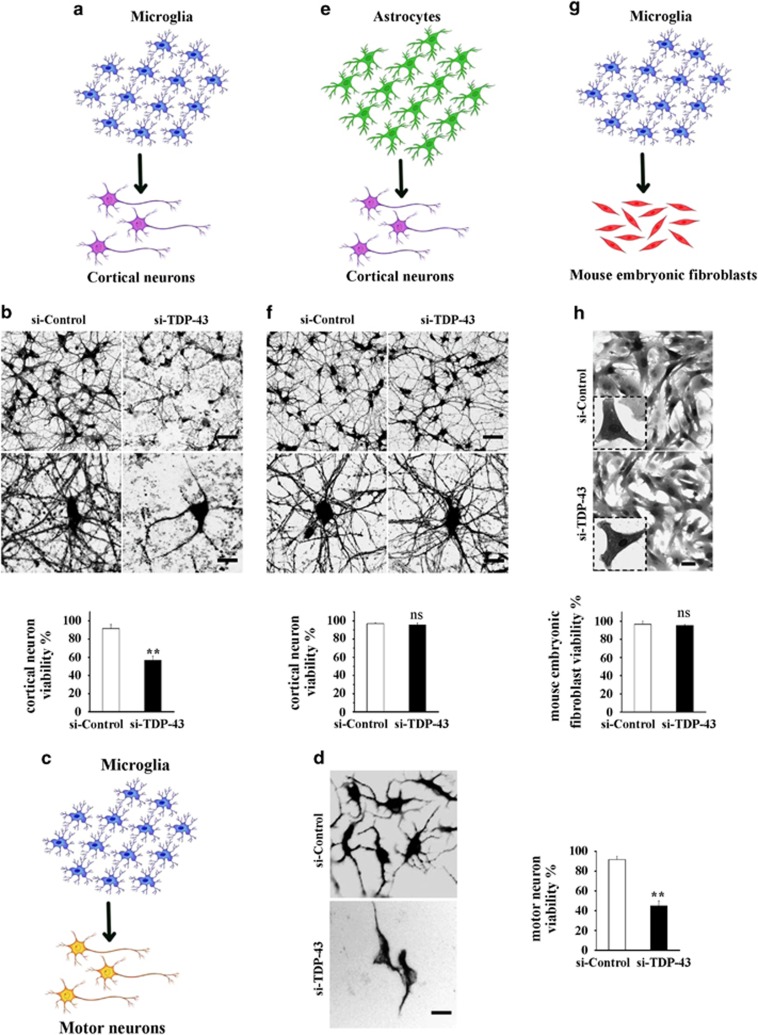
The culture medium from TDP-43-depleted microglia, but not TDP-43-depleted astrocytes, is toxic to neurons. (**a**) Scheme of the conditioned medium assay using TDP-43-depleted primary cultured microglia and cortical neurons. (**b**) Primary cultured microglia were transfected with si-control and si-TDP-43 for 96 h. The culture medium from the last 24 h was harvested and used to culture primary cortical neurons transfected with lentiviral EGFP for 16 h. Then, the cells were visualized using confocal microscopy (Zeiss LSM 710). Scale bars, 30 *μ*m, upper panel; 10 *μ*m, lower panel. The percentage of viability among cortical neurons is shown on the lower side. The data from three independent experiments are presented as the means±S.E.M.; **P<0.01; one-way ANOVA. (**c**) Scheme of primary cultured microglia culture medium treatment of primary cultured motor neurons. (**c** and **d**) Similar experiments as in (**a** and **b**) were performed in primary cultures of motor neurons instead of cortical neurons. The cells were fixed, stained with an antibody against MAP2 (neuronal marker) and visualized using fluorescence microscopy (Olympus IX71). Scale bar, 10 *μ*m. The percentage of motor neurons that were viable is shown on the right side. The data from three independent experiments are presented as the means±S.E.M.; **P<0.01; one-way ANOVA. (**e** and **f**) Similar experiments as in (**a** and **b**) were performed, using primary cultured astrocytes instead of primary cultured microglia. The cells were visualized using confocal microscopy. Scale bars, 30 *μ*m, upper panel; 10 *μ*m, lower panel. The percentage of cortical neurons that were viable is shown on the lower side. The data from three independent experiments indicated the means±S.E.M., ns, not significantly different, one-way ANOVA. (**g** and **h**) Similar experiments as in (**a** and **b**) were performed, using mouse embryonic fibroblasts instead of primary cortical neurons. The cells were visualized using confocal microscopy. Scale bars, 30 *μ*m, upper panel; 10 *μ*m, lower panel. The percentage of primary mouse embryonic fibroblasts that were viable is shown on the lower side. The data from three independent experiments are presented as the means±S.E.M.; ns, not significantly different; one-way ANOVA

Based on the previous observations that increased levels of PGE2 were toxic to neurons, we hypothesized that loss of TDP-43 in microglia could initiate neuronal death owing to the overproduction of PGE2. To test this possibility, we cultured neurons in media collected from TDP-43-depleted microglia (conditioned medium assay) (Figures 5a and c), and the conditioned medium significantly induced cell death in both cortical neurons (Figures 5b and Supplementary Figure S3A) and motor neurons (Figures 5d and Supplementary Figure S3B), but not in mouse embryonic fibroblasts (Figures 5g and h and Supplementary Figure S3C). Interestingly and importantly, the culture medium from TDP-43-depleted astrocytes had no effect on neuron viability (Figures 5e and f).

### Celecoxib alleviates neuronal death induced by TDP-43-depleted microglia

**Figure 6:**
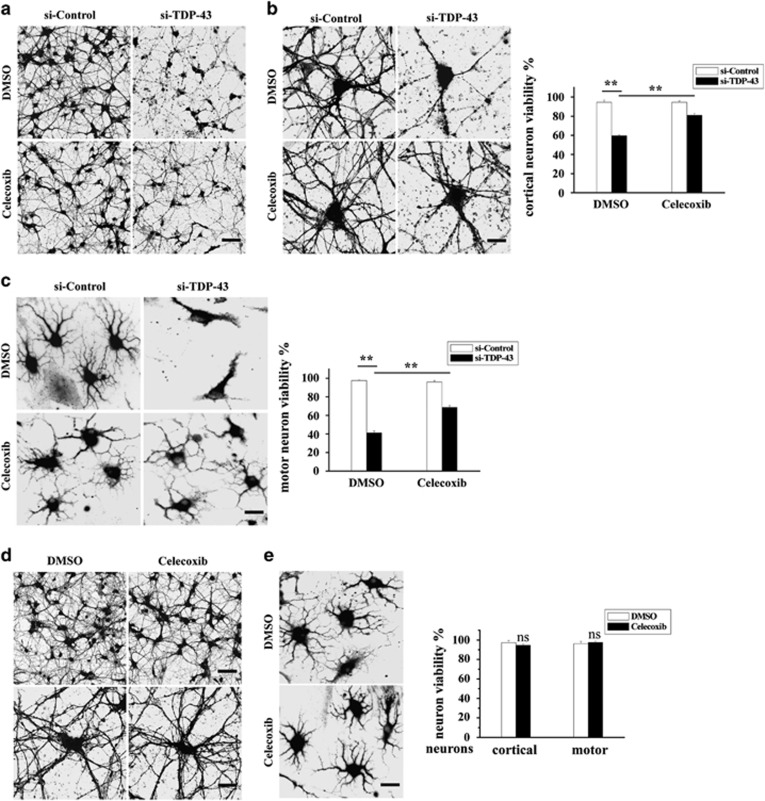
Celecoxib alleviates neuronal death mediated by TDP-43-deficient microglia. (**a** and **b**) BV2 cells were transfected with si-control and si-TDP-43. After 72 h, BV2 cells were treated with DMSO or celecoxib for another 24 h. The culture media from the last 24 h were harvested and used to culture primary cortical neurons transfected with lentiviral EGFP for 8 h (the conditioned medium assay as described for Figure 5). Then, the cells were visualized using confocal microscopy. Scale bars, 30 μm (**a**); 10 μm (**b**). The percentage of cortical neurons that were viable is shown on the right side. The data from three independent experiments are presented as the means±S.E.M.; **P<0.01; one-way ANOVA. (**c**) Similar experiments as in (**a** and **b**) were performed in primary cultured motor neurons. The cells were fixed, stained with an antibody against MAP2 and visualized using fluorescence microscopy. Scale bar, 10 μm. The percentage of motor neurons that were viable is shown on the right side. The data from three independent experiments are presented as the means±S.E.M.; **P<0.01; one-way ANOVA. (**d**) Primary cultured cortical neurons were transfected with lentiviral EGFP. After 72 h, the cortical neurons were treated with DMSO or celecoxib for another 24 h. Then, the cells were visualized using confocal microscopy. Scale bars, 30 μm, upper panel; 10 μm, lower panel. The percentage of cortical neurons that were viable is shown on the right side. The data from three independent experiments are presented as the means±S.E.M.; ns, not significantly different; one-way ANOVA. (**e**) Similar experiments as in (**d**) were performed in primary cultured motor neurons instead of primary cultured cortical neurons. The cells were fixed, stained with an antibody against MAP2, and visualized using fluorescence microscopy. Scale bar, 10 μm. The percentage of motor neurons that were viable is shown on the right side. The data from three independent experiments are presented as the means±S.E.M.; ns, not significantly different; one-way ANOVA

We next investigated whether the COX-2 inhibitor celecoxib could alleviate neuronal death driven by TDP-43-depleted microglia. We performed the conditioned medium assay as described in Figures 5a and c, and we found that the impaired viability of both cortical neurons and motor neurons were restored upon incubating TDP-43-depleted microglia with celecoxib (Figures 6a and c). Meanwhile, celecoxib alone had no effect on the viability of cortical or motor neurons (Figures 6d and e).

## Discussion

**Figure 7:**
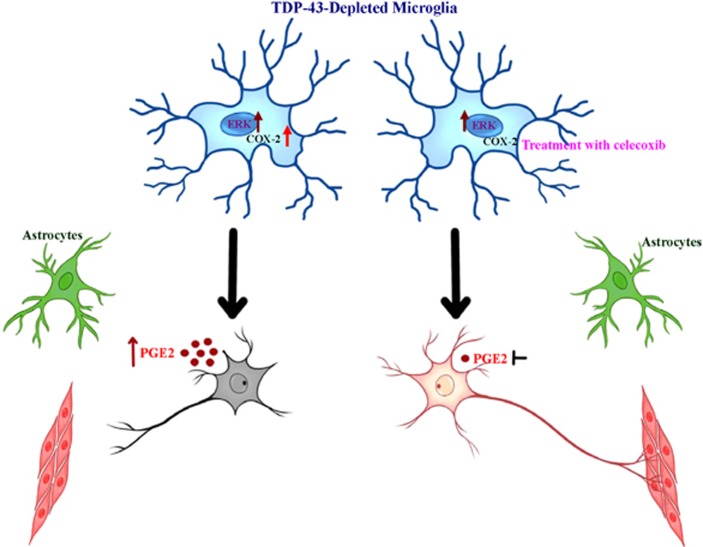
Neuronal death is mediated by the classical MAPK signaling pathway and upregulated PGE2 in TDP-43-depleted microglia. The schematic illustrates the mechanism by which TDP-43-depleted microglia, but not TDP-43-depleted astrocytes, induce neuronal death. The MAPK/ERK signaling pathway is activated in TDP-43-depleted microglia, resulting in increased COX-2 and PGE2, which then trigger neuronal death. Inhibition of COX-2 and PGE2 production using celecoxib alleviates neuronal death mediated by TDP-43-depleted microglia

Although initially, most studies on ALS focused on the selective loss of motor neurons themselves, increasing efforts to understand the role of glial cells in disease pathogenesis have come to the forefront of the field. The present study reveals microglia-mediated COX-2-PGE2 production as the molecular determinant of TDP-43-associated neurotoxicity, emphasizing the important contribution of microglia to non-cell-autonomous motor neuron degeneration in TDP-43-linked ALS (Figure 7).

Although numerous reports have shown that astrogliosis is associated with disease progression, ALS astrocytes carrying pathogenic mutant TDP-43 did not exhibit toxicity to motor neurons in the short term.^51^ On the basis of this finding, it is possible that other types of cells surrounding motor neurons are involved in the initial toxic effect of motor neurons. Consistent with this notion, microglia have recently been suggested to have a role in ALS initiation,^52^ but the contribution of microglia to TDP-43-linked ALS is still not fully understood. In this study, we show that loss of TDP-43 in microglia strikingly triggers the increase of COX-2 (roughly threefold, Figure 1a), accelerating the microglial inflammatory reaction through the release of PGE2 (roughly 10-fold, Figure 4), and mediates selective neuronal death (Figure 5). Because the level of COX-2 in astrocytes is much lower than that in microglia (Figures 1a and b, shown in the immunoblots), we reason that in astrocytes, it would be difficult to increase the expression of COX-2 to levels comparable to those in microglia through the loss of TDP-43, thus, the production of PGE2 in astrocytes would not be sufficient to cause neuronal death (Figure 1b,4b and 5f). Despite that, an interesting study showed that astrogliosis could induce motor neuron death in a rat model with selective overexpression of mutant TDP-43 in astrocytes,^47^ we used a TDP-43 loss-of-function model instead of a TDP-43 gain-of-function model in the current study, and our data showed that loss of TDP-43 in astrocytes is not sufficient to enhance COX-2-PGE2 production, and is thus not sufficient to trigger neurotoxicity. It is therefore possible that the regulation of neuroinflammation may vary in different TDP-43-linked ALS models. Interestingly and importantly, a very recent study provides evidence that microglia specifically induced neurotoxicity via NF-*κ*B activation in a mutant SOD1 transgenic mouse model of ALS. In addition, inhibition of NF-*κ*B in microglia, but not astrocytes, rescued the survival of motor neurons *in vitro* and extended survival in SOD1 mice.^53^ Together with our study, this suggests that microglia may have a key, non-cell-autonomous role in ALS pathogenesis, and it provides novel insight into potential therapeutic treatment of ALS by targeting microglia. Although further investigations are needed to better understand the role of microglia in TDP-43-mediated ALS using better models such as animal models or iPSC-derived cells from ALS patients, a remarkable concordance between the gene expression profile of *in vitro* co-cultured astrocytes with motor neurons carrying mutant SOD1 and spinal cords of mutant SOD1 transgenic mice has been found in a recent study,^54^ suggesting that our study using cultured cell model is highly relevant to *in vivo* ALS research.

Because cytoplasmic TDP-43 aggregates accompanied by a loss of nuclear TDP-43 have been found in ALS patients, a major unresolved question regarding TDP-43-mediated neurodegeneration is that whether the toxicity is triggered by a toxic gain-of-function or by a loss-of-function. Consistent with a gain-of-function mechanism, several cellular signaling pathways, such as PTEN, insulin/IGF-1 and redox signaling, have been reported to regulate TDP-43 in models expressing mutant TDP-43;^55, 56, 57, 58^ consistent with a loss-of-function mechanism, TDP-43 participates in the regulation of the heme oxygenase-1, Rac1-AMPAR and JNK pathways.^59, 60, 61^ However, there is increasing evidence that loss-of-function, rather than gain-of-function, is the major mechanism mediating TDP-43 neuropathology.^5, 12, 13, 14^ Thus, here, we analyzed multiple cellular signaling pathways, including MAPK, JNK, p38 and GSK3*β*, in TDP-43-depleted cells, and we confirmed that MEK-ERK signaling was specifically upregulated in microglia with TDP-43 knockdown (Figures 2a and b). Given that COX-2 expression is controlled by NF-*κ*B and AP-1, two transcription factors that function downstream of MEK-ERK signaling,^62, 63^ we thought to test whether NF-*κ*B or AP-1 was involved in TDP-43-mediated regulation of COX-2. Our data indicate that NF-*κ*B was not involved in this regulation because blocking NF-*κ*B activity does not change the effect of TDP-43 on COX-2 expression (Figure 3a). Although a previous study showed that TDP-43 is associated with NF-*κ*B activation and inflammation,^64^ it should be noted that TDP-43 itself did not regulate NF-*κ*B activation and inflammation in their observations.^64^ It is possible that other inflammatory inducers and stimuli may help to trigger NF-*κ*B-mediated inflammation in TDP-43-depleted microglia. In the current study, we find that TDP-43 can directly regulate COX-2-PGE2 production (without extra stimuli), indicating that signaling molecules other than NF-*κ*B are required for this regulation. Relatedly, our results show that knockdown of TDP-43 in microglia resulted in the activation of AP-1 (Figure 3b) and led to marked increases in both PGE2 and COX-2. Moreover, inhibition of MEK-ERK signaling by U0126 strikingly diminished the abnormal increases in AP-1 activity, COX-2 expression and PGE2 production in TDP-43-deficient microglia (Figures 2d,3c and 4c). Taken together, these data reveal that the abnormal activation of MEK-ERK-AP-1 signaling is directly associated with the upregulation of COX-2-PGE2 production in TDP-43-depleted microglia.

We found increased COX-2 expression and PGE2 production in TDP-43-deficient microglia (Figures 1a and 4a), and inhibition of COX-2 expression and PGE2 production by celecoxib treatment reduced the neurotoxicity triggered by TDP-43-deficient microglia (Figures 6a and c). Consistent with our observations, previous studies have shown that inhibition of COX-2 and PGE2-mediated inflammation was therapeutically effective in mutant SOD1 transgenic mice.^28, 65^ Given that abnormal TDP-43 function is tightly associated with most ALS cases, the present study provides novel insight into how microglia induce neurotoxicity in ALS, and, more interestingly, suggests celecoxib as a potential therapy for ALS.

## Materials and Methods

### Antibodies

The anti-TDP-43 antibody was described previously,^66^ and the following additional primary antibodies were used: anti-iNOS (Abcam, Cambridge, MA, USA), anti-COX-2 (Epitomics, Burlingame, CA, USA), anti-GAPDH (Chemicon, Temecula, CA, USA), anti-phospho-MEK (S217/221) (Cell Signaling Technology, Beverly, MA, USA), anti-MEK (Santa Cruz Biotechnology, Santa Cruz, CA, USA), anti-phospho-ERK (Santa Cruz Biotechnology), anti-ERK (Santa Cruz Biotechnology), anti-phospho-JNK (Epitomics), anti-JNK (Santa Cruz Biotechnology), anti-phospho-p38 (Epitomics), anti-p38 (Santa Cruz Biotechnology), anti-phospho-c-Raf (S338) (Cell Signaling Technology), anti-c-JUN (Proteintech, Chicago, IL, USA), anti-phospho-Glycogen Synthase (S641) (p-GS) (Epitomics) and anti-MAP2 (Santa Cruz Biotechnology). The secondary antibodies used included horseradish peroxidase-conjugated sheep anti-mouse and anti-rabbit antibodies (Amersham Pharmacia Biotech, Peapack, NJ, USA). The antibody-bound proteins were visualized using an ECL detection kit (Amersham Biosciences, Piscataway, NJ, USA) or Alexa Fluor 488 (green) donkey anti-mouse IgG (Invitrogen, La Jolla, CA, USA).

### Cell culture, transfection, drug treatment, and RNAi and cell viability

Mouse motor neuron cell line (NSC-34 cells) were cultured in Dulbecco's modified Eagle's medium (DMEM) (Gibco, Grand Island, NY, USA) containing 10% fetal bovine serum (FBS) (Gibco) with penicillin (100 mg/ml) and streptomycin (100 mg/ml). Mouse microglial cell line (BV2 cells) were cultured in similar culture medium, but using 10% heat-inactivated FBS instead. For RNAi experiments, cells were transfected with siRNA against TDP-43 using the RNAiMAX (Invitrogen) transfection reagent according to the manufacturer's instructions. Subsequently, cells were treated for 24 h with DMSO (Sangon Biotech, Shanghai, China) or U0126 (Beyotime, Shanghai, China) (20 *μ*M), BAY (Beyotime) (25 *μ*M), SP600125 (10 *μ*M), SB216763 (Sigma, St. Louis, MO, USA) (10 *μ*M), SB203580 (10 *μ*M), celecoxib (Sigma) (50 *μ*M), or PBS (Gibco) or LPS (Beyotime) (0.1 *μ*g/ml). The following sequences were used for siRNA targeting mouse TDP-43 (si-TDP-43): 5′-GGATCTGAAAGACTATTTC-3′ siRNA targeting rat TDP-43 (si-TDP-43): 5′-CCAATGCTGAACCTAAGCA-3′. For cell viability analysis, the cells were stained with PI and visualized using fluorescence microscopy. The dead cells with PI-positive staining were counted and the percentage of viability of cells was quantified.

### Primary culture of microglia, astrocytes, cortical neurons, spinal cord motor neurons and mouse embryonic fibroblasts

Primary cultured microglia were prepared as described elsewhere.^67^ In brief, the cortex of new born SD rats was chopped and dissected. Cortex tissues were incubated with 0.25% Trypsin (Gibco) for 15 min at 37 °C, and then the cells were dissociated with a plastic pipette. Subsequently, cells isolated from cortex tissue were plated on poly-D-lysine-coated 24-well plates (Corning, Tewksbury, MA, USA) and cultured at 37 °C, 5% CO2. Mixed glial cells were cultured in DMEM/F12 with 10% heat-inactivated FBS and penicillin (100 mg/ml) and streptomycin (100 mg/ml), and the culture medium were replaced every 3 days. After 14 days, microglia were separated from mixed glial cells by shaking at 150 rpm for 2 h at 37 °C. Primary cultured astrocytes were similarly dissociated from the cortex of new born ICR mice and cultured in DMEM with 10% FBS and penicillin (100 mg/ml) and streptomycin (100 mg/ml). Cortical neurons were similarly dissociated from the cortex of ICR mouse embryos at embryonic day 17 (E17), and motor neurons were similarly dissociated from the spinal cord of ICR mouse embryos at E17 as previously described.^68^ In brief, the dissociated neurons were cultured in neurobasal medium (Gibco) with 10% FBS, 1 × B27 (Gibco) and glutamine (0.05 mg/ml; Sigma) for 12 h. Subsequently, culture medium were change to neurobasal medium with 1 × B27 and glutamine (0.05 mg/ml). After 5 days, the neurons were subjected to experiments. The purity of motor neurons were shown in Supplementary Figure S4A using specific markers of neurons (MAP2) and astrocytes (GFAP). Mouse embryonic fibroblasts were obtained from the epithelium of ICR mouse embryos at E17, and cultured as described for primary cultured astrocytes.

### Immunoblot

Cells were harvested and lysed in cell lysis buffer (50 mM Tris-HCl (pH 7.6) with protease inhibitor cocktail (Roche, Indianapolis, IN, USA), 150 mM NaCl, 0.5% sodium deoxycholate, and 1% Nonidet P-40). Then, the proteins were separated by 10% or 12% SDS-PAGE (polyacrylamide gel electrophoresis) and transferred onto polyvinylidene difluoride membranes (Millipore, Bedford, MA, USA).

### QRT-PCR

Total RNA from BV2 cells was extracted with TRIzol Reagent (Invitrogen); subsequently, the RNA was reverse-transcribed into cDNA using PrimeScript RT Master Mix (Takara, Shiga, Japan). Real-time PCR analysis was performed using SYBR Green Real-Time PCR Master Mix (Takara) using a CFX96 Real-Time System (BIO-RAD, Hercules, CA, USA) and the following primers: mouse *β*-actin: 5′-GACCTGACTGACTACCTC-3′ and 5′-GACAGCGAGGCCAGGATG-3′, mouse iNOS: 5′-TCCCAGCCTGCCCCTTCAAT-3′ and 5′-CGGATCTCTCTCCTCCTGGG-3′, and mouse COX-2: 5′-CAGGCTGAACTTCGAAAC A-3′ and 5′-GCTCACGAGGCCACTGATACCTA-3′.

### Analysis of PGE2 production

Primary cultured microglia and BV2 cells were transfected with siRNA against TDP-43. After transfection, the cells were treated with U0126 or celecoxib for 24 h, and then the levels of PGE2 in 50 *μ*l of 400 *μ*l medium were measured with PGE2 ELISA kits (Cayman Chemical Company, Ann Arbor, MI, USA) according to the manufacturer's instructions.

### Luciferase reporter gene assay

BV2 cells were plated on 24-well plate and incubated for 16 h at 37 °C. Then the cells were transfected with Cignal lentiviral AP-1 Reporter (luc) (QIAGEN, Hilden, Japan) according to the manufacturer's instructions for 24 h. Subsequently, the culture medium were replaced with fresh medium. After another 24 h, the cells were cultured in culture medium supplement with 2.5 *μ*g/ml of puromycin. After 5 days, survived BV2 cells were selected and the BV2 stable cell line expressing AP-1 reporter construct was generated. Subsequently, the BV2 cells stably expressing AP-1 constructs were transfected with siRNA against TDP-43. After 48 h, the cells were treated with U0126 for 24 h, and then the cells were harvested. Finally, the AP-1 promoter activity was measured using the luciferase assay kit (Promega, Madison, WI, USA) according to the manufacturer's instructions.

## Abbreviations

ALS: amyotrophic lateral sclerosis
AD: Alzheimer's disease
COX-2: cyclooxygenase-2
DMSO: dimethyl sulfoxide
EGFP: enhanced green fluorescent protein
FBS: fetal bovine serum
GAPDH: glyceraldehyde-3-phosphate dehydrogenase
FTLD: frontotemporal lobar degeneration
MAPK: mitogen-activated protein kinase
PGE2: prostaglandin E2
PD: Parkinson's disease
qRT-PCR: quantitative real-time PCR
siRNA: small interfering RNA
*SOD1*: Cu/Zn superoxide dismutase 1
*TARDBP*: TAR DNA-binding protein 43
