# Supplementary material for: Induction of COX-2-PGE2 synthesis by activation of the MAPK/ERK pathway contributes to neuronal death triggered by TDP-43-depleted microglia
Source: Cell Death Dis. 2015 Mar 26;6(3):e1702–. doi: 10.1038/cddis.2015.69 (PMC4385945; doi:10.1038/cddis.2015.69)
Supplement: Supplementary Figures [file cddis201569x1.pdf]

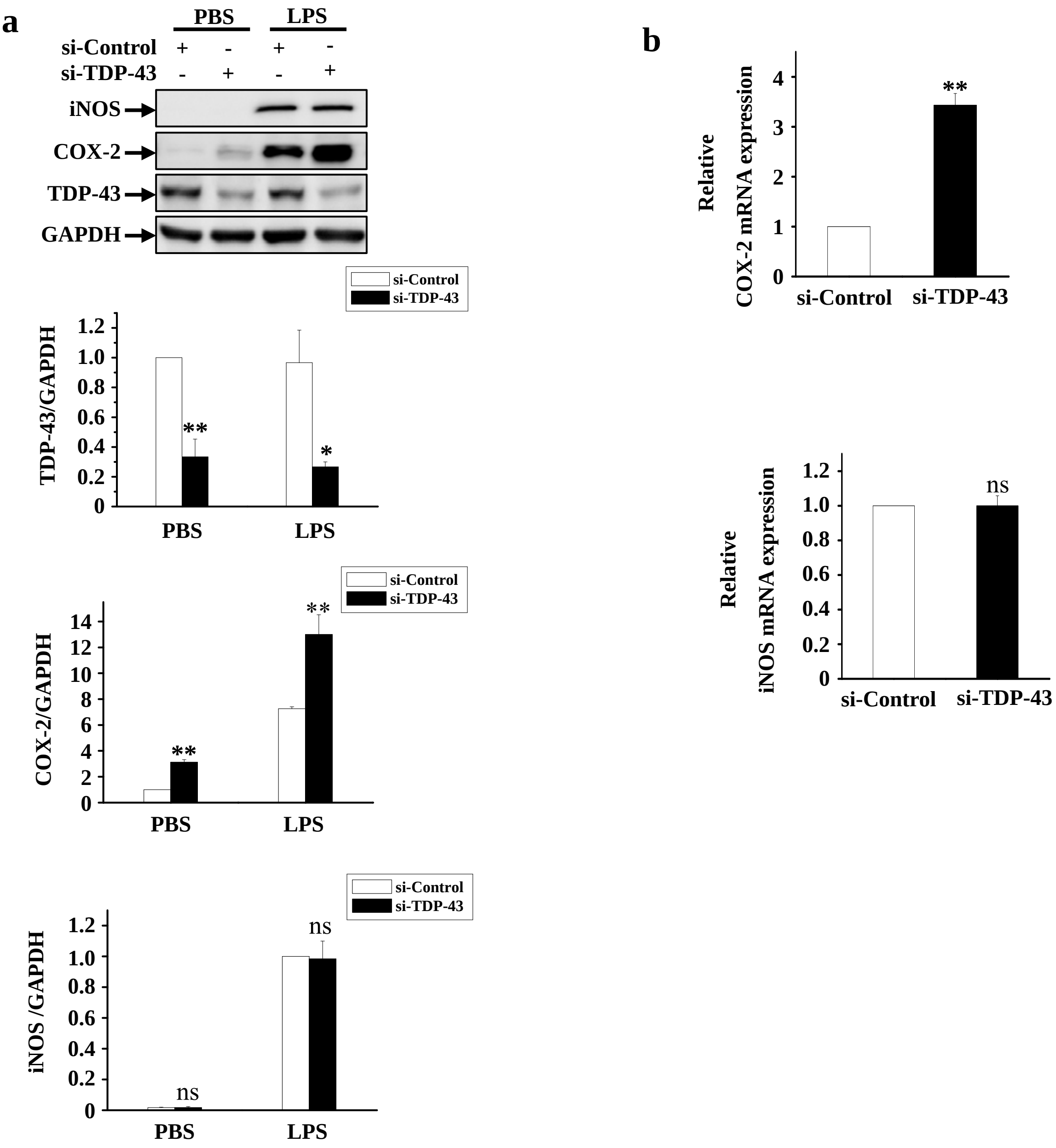

Supplementary Figure 1. TDP-43 specifically regulates the expression level of COX-2, but not iNOS, in BV2 cells, related to figure 1. (a) BV2 cells were transfected with si-Control and si-TDP-43. After 48 h, the cells were treated with PBS or LPS (0.1 μg/ml) for 24 h, and then the cell lysates were subjected to immunoblot analysis using antibodies targeting iNOS, COX-2, TDP-43 and GAPDH. The quantification of TDP-43, COX-2 and iNOS levels is shown in the lower panels, representing three independent experiments. The data are presented as the means ± S.E.M.; ns, not significantly different; *, $p<0.05$; **, $p<0.01$; one-way ANOVA. (b) BV2 cells were transfected with si-Control and si-TDP-43 and cultured for 72 h, and then the cells were processed for qRT–PCR. The mRNA levels of COX-2 and iNOS were each quantified and normalized to GAPDH. The data from three independent experiments are shown as the means ± S.E.M., ns, not significantly different; **, $p<0.01$; one-way ANOVA.

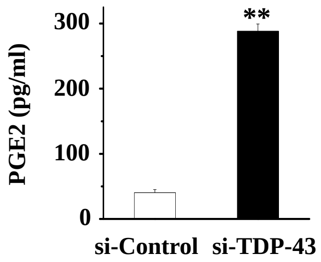

Supplementary Figure 2. Loss of TDP-43 strikingly promotes the production of PGE2 in BV2 cells but not in astrocytes, related to figure 4. BV2 cells were transfected with si-Control and si-TDP-43 for 72 h at a final density of 35 × 10^4 cells per 24-well. Then, the media from for 24 h cultures of BV2 cells and subjected to PGE2 enzyme-linked immunosorbent assays (ELISAs). The data from three independent experiments are presented as the means ± S.E.M.; **, $p<0.01$; one-way ANOVA.

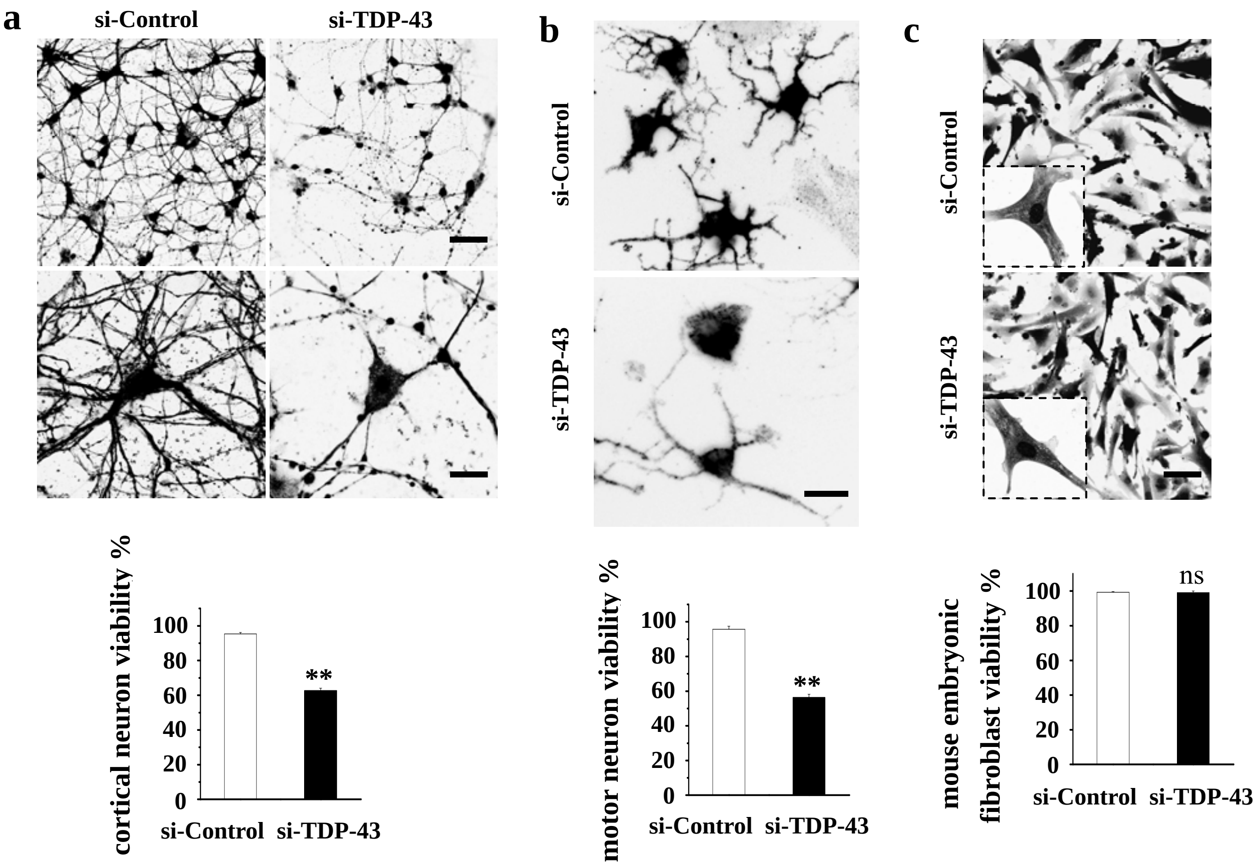

Supplementary Figure 3. The culture medium from TDP-43-depleted BV2 is toxic to neurons, related to figure 5.

(a) BV2 cells were transfected with si-Control and si-TDP-43 for 96 h. The culture medium from the last 24 h was harvested and used to culture primary cortical neurons transfected with lentiviral EGFP for 8 h. Then, the cells were visualized using confocal microscopy. Scale bars, 30 μm, upper panel; 10 μm, lower panel. The percentage of viability among cortical neurons is shown on the lower side. The data from three independent experiments are presented as the means ± S.E.M.; **, $p<0.01$; one-way ANOVA. (b) Similar experiments as in (a) were performed in primary cultures of motor neurons instead of cortical neurons. The cells were fixed, stained with an antibody against MAP2 (neuronal marker), and visualized using fluorescence microscopy. Scale bar, 10 μm. The percentage of motor neurons that were viable is shown on the lower side. The data from three independent experiments are presented as the means ± S.E.M.; **, $p<0.01$; one-way ANOVA. (c) Similar experiments as in (a) were performed, using mouse embryonic fibroblasts instead of primary cortical neurons. The cells were visualized using confocal microscopy. Scale bar, 10 μm. The percentage of primary mouse embryonic fibroblasts that were viable is shown on the lower side. The data from three independent experiments are presented as the means ± S.E.M.; ns, not significantly different; one-way ANOVA.

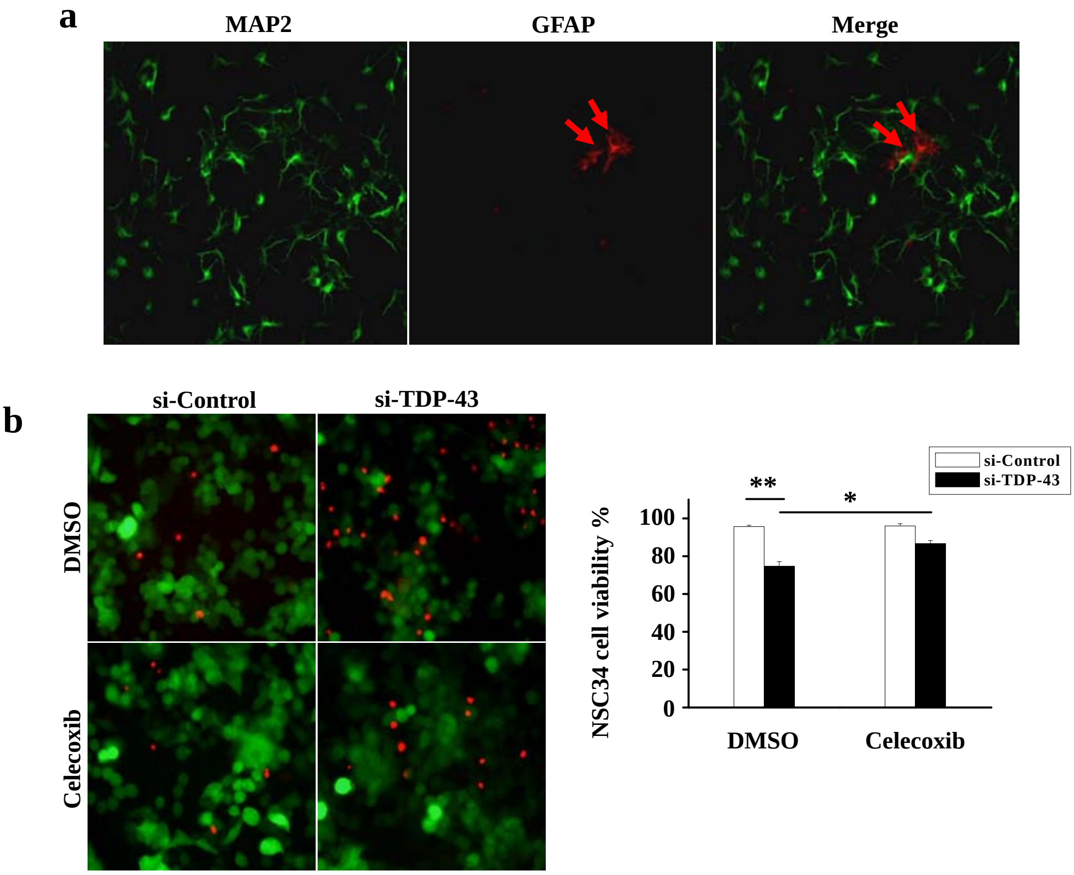

Supplementary Figure 4. Celecoxib alleviates neuronal death mediated by TDP-43 deficient microglia, related to figure 6.

(a) Primary cultured motor neurons were fixed, stained with antibodies against MAP2 (neuronal marker) and GFAP (astroglial marker), and visualized using fluorescence microscopy. Red arrows indicated astrocytes. Our data indicate that the majority of the culture cells are motor neurons (more than 95%). (b) Primary cultured microglia were transfected with si-Control and si-TDP-43 for 96 h, and then were treated with DMSO or celecoxib for another 24 h. Subsequently, the culture media were harvested and used to culture NSC-34 cells transfected with lentiviral EGFP. After 24 h, the cells were stained with PI and visualized using fluorescence microscopy. The dead cells with PI-positive staining were counted and the percentage of viability of NSC34 cells is shown on the right side. The data from three independent experiments are presented as the means ± S.E.M.; *, $p<0.05$; **, $p<0.01$; one-way ANOVA.
